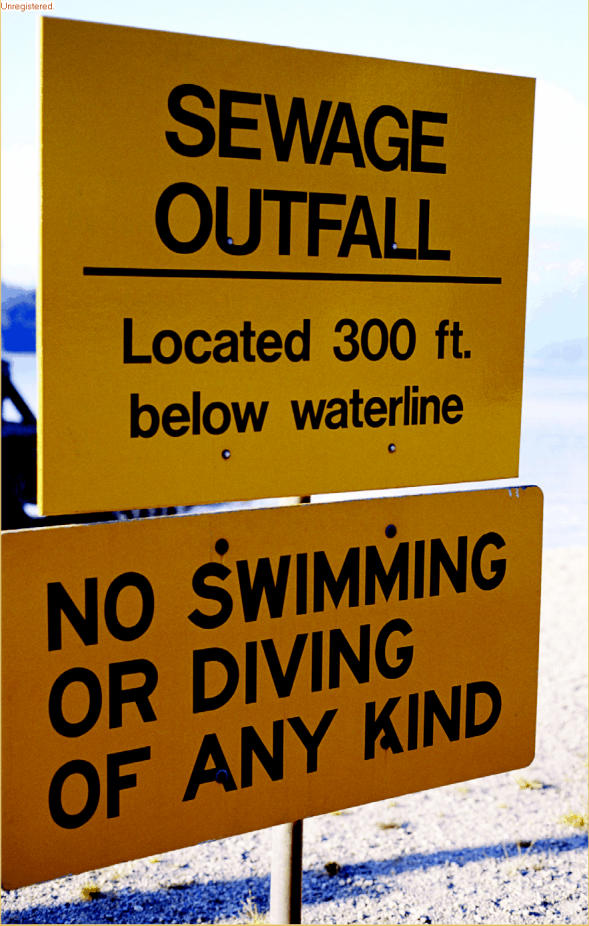# Combined Sewer Systems: Down, Dirty, and Out of Date

**DOI:** 10.1289/ehp.113-a464

**Published:** 2005-07

**Authors:** John Tibbetts

When combined sewer systems were introduced in 1855, they were hailed as a vast improvement over urban cesspool ditches that ran along city streets and spilled over when it rained. These networks of underground pipes were designed to dry out streets by collecting rainwater runoff, domestic sewage from newly invented flush toilets, and industrial waste-water all in the same pipe. Waste- and stormwater was then discharged directly into waterways; in the early twentieth century, sewage treatment plants were added to clean the wastewater before it hit streams. Combined sewer systems were—and still are—a great idea, with one catch: when too much stormwater is added to the flow of raw sewage, the result is frequently an overflow. These combined sewer overflows (CSOs) have become the focus of a debate regarding the best techniques to manage growing volumes of sewage and stormwater runoff in many older U.S. communities.

In dry weather, a combined sewer system sends a town’s entire volume of waste-water to a sewage plant, which treats and discharges it into a waterway. Rain and snowmelt, however, can fill up a combined sewer. The sewers have been specifically designed with escape overflow pipes so that the mixture of sewage and stormwater doesn’t back up into buildings, including homes. The resulting CSO dumps raw sewage into lakes, rivers, and coastal waters, potentially harming public health and the environment.

In April 1994, the U.S. Environmental Protection Agency (EPA) issued the CSO Control Policy, the national framework for control of CSOs, through the National Pollutant Discharge Elimination System permitting program. This policy mandated that communities dramatically reduce or eliminate their CSOs, and the agency began working with municipalities to improve antiquated sewage systems so they could reach Clean Water Act goals. Under this policy, communities with combined sewer systems must establish a short-term plan to control these discharges as well as a long-term control plan.

The EPA’s mandate on CSOs leaves communities with two basic options, according to Joan B. Rose, a public health microbiologist at Michigan State University. Communities with CSOs can build separate underground pipes for sewage and stormwater. Or they can keep their combined pipes and somehow build more capacity. “But if they shut down [combined sewer systems],” she says, “communities must find a way to store or treat peak flows when it rains.”

## When CSOs Occur

About 40 million people in 32 states live in cities with combined sewer systems; most of these systems are found in Maine, New York, Pennsylvania, West Virginia, Ohio, Indiana, Michigan, and Illinois. CSOs are a major water pollution concern for 772 cities, according to the EPA’s 2004 *Report to Congress: Impacts and Control of CSOs and SSOs* [sanitary sewer overflows, which are associated with another type of sewer system].

Although some major cities like New York City and Philadelphia have combined sewer systems, most communities with CSO problems have fewer than 10,000 people, according to the EPA report. One reason lies in the economy of scale: larger municipalities are more likely to have sufficient tax base and water users to finance remedies to CSO problems.

CSOs annually result in an estimated 850 billion gallons of untreated wastewater and stormwater being discharged into U.S. waterways, according to the EPA report. Thanks to the CSO Control Policy, this is an improvement over figures in the agency’s 2001 report on the same topic, which put the figure at 1.3 trillion gallons per year.

CSOs flood waterways with contaminants including microbial pathogens, suspended solids, chemicals, trash, and nutrients that deplete dissolved oxygen. Microbial pathogens and toxics can be present in CSOs at levels that pose risks to human health. CSOs can therefore lead to contamination of drinking water supplies, beaches, and shellfish beds.

The EPA’s 2004 report indicated that the agency has limited information on the extent of human health impacts of CSOs. One health effect the agency can quantify comes from regularly monitored coastal and Great Lakes beaches. Using data from these locations, the EPA estimates about 3,500–5,500 gastrointestinal illnesses each year are caused by CSO and SSO pollution of swimming waters.

According to Rose, current estimates hold that microbial pathogens in U.S. public drinking water supplies sicken hundreds of thousands of people each year, though most of these waterborne illnesses are mild, disappearing after a few days. It’s difficult, moreover, to trace sources of these illnesses. Many outbreaks in the United States go unreported, and in most outbreaks the pathogen is not identified. CSOs may or may not be to blame.

However, several reports and studies, including one from the 22 November 2002 *Morbidity and Mortality Weekly Report*, demonstrate that there has been an increase in waterborne disease outbreaks in the United States over the past few years. Rose says these community outbreaks are correlated with rainfall as well as associated overflows and leaks in public sewer systems. Sensitive populations—the elderly, the very young, and those with existing health problems—are most vulnerable to waterborne enteric microorganisms. These populations make up about 20% of the U.S. public.

## A Controversial Alternative

In most municipal treatment plants, waste-water usually goes through a two-step process before it is discharged into lakes, rivers, and coastal waters. Large solids are removed first during primary treatment—mechanical screens remove large debris, and sedimentation tanks remove sludge (solids that sink) and scum (elements that rise to the top). In the second step, wastewater is first routed to tanks with activated microbes that break down organic materials and remove some pathogens and more of the remaining solids. This biological treatment can improve the effectiveness of disinfection, which is often the second part of this step—chlorine is used to kill bacteria and other remaining pathogens before the water is released.

This treatment process is the most effective way to ensure that effluent is clean. It has become the required standard for wastewater treatment under the Clean Water Act. But many plants have smaller biological treatment capacities than primary treatment capacities. Biological treatment facilities are expensive, and many communities have outgrown systems that were built 30–40 years ago. Moreover, these facilities can be delicate. Large waste-water flows into biological treatment units, such as those following heavy rains, can wash the microorganisms from the tanks. The units must then be shut down until the microbial population replenishes itself.

“Blending,” or “bypassing,” is one engineering technique that many sewerage operators have used to handle peak flows. During wet weather, utilities route a portion of peak wastewater flows around the biological treatment units, then combine the rerouted flows with the portion of wastewater that went through biological treatment. After blending, the effluent is usually disinfected and discharged into water bodies.

For decades, environmental permitting agencies in some states have allowed sewerage operators to use this technique in an effort to avoid CSOs. Recently, however, blending has become a controversial practice debated by the wastewater industry, environmentalists, and public health scientists.

The wastewater treatment industry argues that bypassing biological treatment for a portion of the water is a significant improvement over releasing completely untreated wastewater, which is what happens when combined sewers overflow. “If the choice is between raw sewage getting sent into waterways and wastewater getting sent to the treatment facility, most people would rather that the wastewater get treatment,” says Alexandra Dunn, general counsel of the National Association of Clean Water Agencies, a trade group representing more than 300 utilities.

However, critics of blending say the process allows for a higher concentration of pollutants to be released into water bodies, potentially sickening more people. When blending wastewater rather than fully treating it, utilities are less effective at removing microbial pathogens, says Charles Haas, an environmental engineer at Drexel University. “It’s much more difficult to disinfect poorly treated wastewater than fully treated wastewater, and I would consider primary-treated wastewater as poorly treated,” he says. Many solids may still remain in primary-treated wastewater, and viruses, parasites, and bacteria within those solids are protected from disinfection, he adds.

“When the wastewater industry talks about blending versus CSOs, they argue that blending is better in terms of protecting public health,” Rose says. “But I don’t think they have the data on pathogens—viruses and parasites—to prove that. Much more research is needed on wastewater and on treatment to control pathogen risks.”

## Toward Better Blending

Nancy Stoner, director of the Clean Water Project at the Natural Resources Defense Council, says that current blending policy as outlined in the CSO Control Policy is chaotic and poorly regulated: “A lot of treatment plants do blending [almost anytime it rains], and some are given the leeway by states to do blending, while other treatment plants are not given the leeway but do it anyway.” In recent years, sewerage operators have sought guidance from the federal government on the blending issue.

In November 2003, the EPA proposed a new federal policy that would have authorized municipal sewage plants around the country to blend wastewater in certain circumstances and under certain conditions—for example, only during periods of heavy rain or snowmelt, and only if plants were already meeting effluent standards required for permitting. The EPA said that its proposed policy was already common practice in many communities.

During the public comment period, the EPA received about 98,000 comments, and the proposal was not warmly embraced by environmentalists. “EPA’s proposal would put more partially treated sewage into the environment,” says Stoner. “The solution to overflows is not to bypass, but to fix the leaky sewer systems.”

Congress reacted, too. On 3 March 2005, members of the House of Representatives introduced bipartisan legislation, the “Save Our Waters From Sewage Act,” to block the EPA blending proposal. The legislation called for amendments to the Clean Water Act to “prohibit a publicly owned treatment works from diverting flows to bypass any more of its treatment facility unless the bypass is unavoidable . . . [and] there is not a feasible alternative to the bypass.”

On 19 May 2005, the EPA announced that it would not finalize the sewage blending policy as proposed in November 2003. “Blending is not a long-term solution,” said Benjamin Grumbles, assistant administrator for the Office of Water, in a press release at the time. “Our goal is to reduce overflows and increase treatment of waste-water to protect human health and the environment.” The agency also said it will continue to review policy and regulatory alternatives to create feasible approaches to treat wastewater.

## The Cost of Fixing Systems

According to the EPA’s 2000 Clean Watersheds Needs Survey, it would cost about $50.6 billion over the next 20 years to reduce the nation’s CSO volume by 85%. In recent years, some communities with CSOs have increased sewer rates to raise funds to upgrade their infrastructure. But it’s difficult for many localities to pay for large-scale sewer and water treatment facilities without federal and state aid, says Dunn. Some relatively prosperous communities such as Grand Rapids, Michigan, are in the process of installing separate stormwater pipes, she adds, but this is not feasible for large, financially distressed cities such as Detroit.

Municipalities, sewerage operators, public health scientists, and environmentalists are calling for more federal funding to replace aging pipes and upgrade treatment systems. But federal spending for sewerage infrastructure is actually falling. During fiscal year 2005, Congress cut $250 million from the Clean Water State Revolving Fund (CWSRF), which provides low-cost loans primarily for sewerage infrastructure upgrades. In President Bush’s fiscal year 2006 budget proposal, this fund faces a further one-third reduction.

Even in the best of fiscal times, the CWSRF, distributed among 50 states, cannot address municipal needs to borrow for CSO projects and repay on favorable terms. “In most cases,” says Dunn, “the total allocation to a state per year could be used by one city alone for one phase of its project.” For example, she says, the first phase of the CSO program in place at Narragansett Bay, Rhode Island, will cost $250 million, which could use up all of Rhode Island’s annual portion of the CWSRF.

Despite falling federal aid, communities still—as mandated by the CSO Control Policy—must establish and find a way to implement long-term control plans that will provide for full compliance with the Clean Water Act, including significant reduction of CSOs. Communities are in various stages of developing and implementing their long-term plans.

Some cities such as Boston, Chicago, and Atlanta have built deep storage tunnels to hold stormwater overflows, says Chris Hornback, regulatory director of the National Association of Clean Water Agencies. Eventually, the extra wastewater can be treated at a flow that works for a particular wastewater plant. “Building these storage tunnels is a simple, straightforward process, but it costs hundred and hundreds of millions of dollars, and at some point you reach a break point in cost,” he says.

Environmentalists call for less costly methods of reducing stormwater runoff and CSOs. Such methods, says Stoner, include better means of trapping storm-water before it reaches sewers and putting it into the ground instead. Installing rain gardens, permeable pavements, roof gardens, or even just grassy swales or ditches along roadways can be beneficial for a number of reasons: soil and vegetation provide filtration, groundwater supplies are replenished, and overland stormwater flows are diminished. Such methods are mostly low-tech and cost-effective. [For more information on reducing runoff, see “Paving Paradise: The Peril of Impervious Surfaces,” p. A456 this issue.]

Even so, low-impact techniques alone will not be enough to fully control the CSO problem, according to the EPA’s 2004 report. Environmentalists and municipalities agree that, contrary to the current trend, the answer will depend on greater federal investment in wastewater infrastructure around the country.

## Figures and Tables

**Figure:**
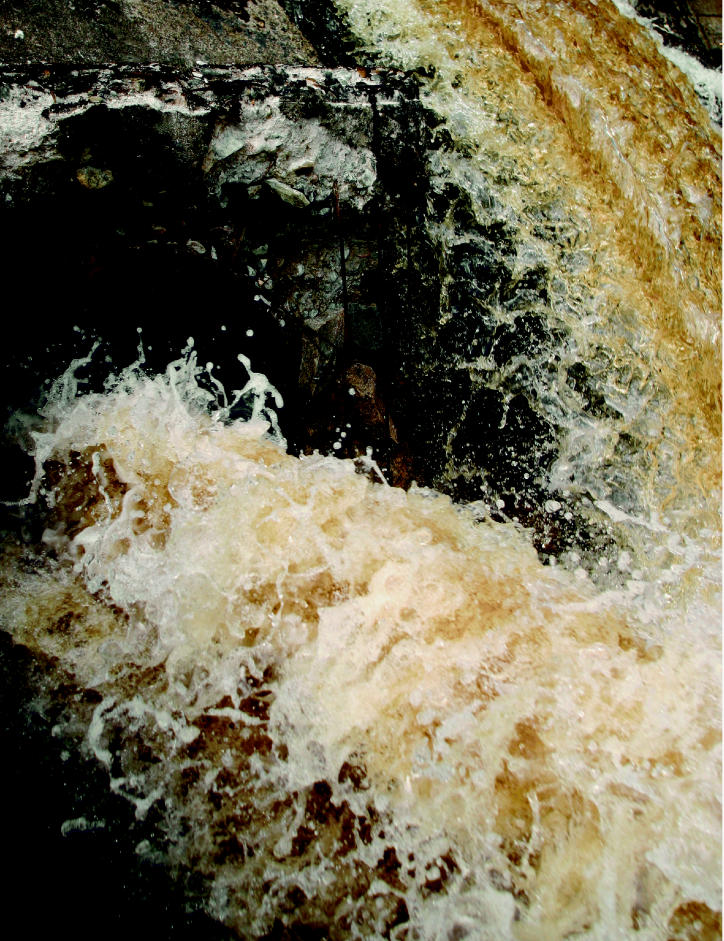


**Figure:**